# The Herschel-Quincke tube with modulated branches

**DOI:** 10.1098/rsta.2022.0074

**Published:** 2022-11-28

**Authors:** T. K. Papathanasiou, E. Tsolakis, V. Spitas, A. B. Movchan

**Affiliations:** ^1^ Department of Civil Engineering, Aston University, Birmingham B4 7ET, UK; ^2^ School of Mechanical Engineering, National Technical University of Athens, Zografou 15773, Greece; ^3^ Department of Mathematical Sciences, University of Liverpool, Liverpool L69 7ZL, UK

**Keywords:** modified Herschel-Quincke tube, fluid-borne noise, transmission loss, modulated waveguides, periodic structures

## Abstract

The Herschel-Quincke (HQ) tube concept for transmission loss in pipe systems is expanded to include cases of branches with modulated properties. Modulated waveguides, featuring corrugations in their geometry or speed of sound, are known to produce significant reflection even without the parallel branch of the HQ tube. The HQ tube, in its classical form, produces narrow banded transmission loss at frequencies related to the length, wavenumber and cross-section area of the parallel branch. The modulated Herschel-Quincke (MHQ) tube combines these attributes to produce enhanced transmission loss characteristics in terms of both width and number of transmission loss bands. Several modulated profiles for the speed of sound in different branches of the tube are considered and analytical expressions for the transmission loss and resonant conditions are derived. Detailed analysis of periodically stratified branch profiles demonstrates the effectiveness of the MHQ tube for fluid-borne noise attenuation in pipe systems.

This article is part of the theme issue 'Wave generation and transmission in multi-scale complex media and structured metamaterials (part 2)'.

## 1. Introduction

Attenuators, such as silencers or pulse damping devices, are integrated in hydraulic and exhaust pipe systems to suppress fluid-borne noise. The Herschel-Quincke tube (HQ), introduced in the nineteenth century by Herschel [1] (1833) and subsequently Quincke [2] (1866), is a silencer based on the concept of destructive interference. Although the HQ tube in its classic form is a relatively simple configuration, consisting of two one-dimensional ducts running in parallel, it features quite complex dynamics. Quantitative analysis of the HQ tube dynamics was first conducted by Stewart [3,4]. In Stewart's analysis, the parallel ducts of the tube were set to have the same cross-section area. This restriction has been shown [5,6] to limit the attenuation effectiveness. Selamet & Dickey [6] presented a study that combined theoretical formulaes, experiments and numerical simulation for an HQ tube with unequal branch cross-section dimensions and demonstrated that broadband attenuation is possible, contrary to the equal cross-section branch approach.

Several authors have introduced and studied configurations that generalize the HQ tube concept, aiming at enhanced, broadband transmission loss characteristics. Selamet & Easwaran [7] studied an HQ tube with an arbitrary number of branches. This development introduced more degrees of freedom to tune resonances and hence more flexibility in designing HQ tube silencers for broadband attenuation. Further developments include analysis of mean flow effects [8], the HQ system with arbitrary number of ducts in the presence of mean flow [9], active variation of parallel branch length and successive HQ tubes in the same circuit [10], use of interconnecting branches [11] and adaptive variation of the parallel branch properties using active membranes or piston elements [12].

In the case of constant and uniform branch properties, the increased Transmission Loss (TL) bands depend on the number of parallel ducts, the cross-section area of each duct and its Helmholtz number, i.e. the product He = *ωL*/*c*, where *ω* is the angular natural frequency, *L* the duct length and *c* the speed of sound [4,7]. Increasing the number of parallel branches to form an *N*-duct HQ tube allows for enhanced attenuation characteristics if appropriate tuning of branch length and cross-section is achieved. Fundamentally, tuning can be achieved either thought changing the branch lengths and cross-sections or the branch celerity. However, there are certain cases where specific constraints render practical solutions impossible. Among several restrictive factors are the lack of space for very elongated branches when several modern exhaust pipe or hydraulic circuit applications are considered (e.g. automotive and aerospace industry applications). Reviewing the definition of the Helmholtz number, it is evident that instead of increasing the branch length reducing sufficiently the celerity could produce the same effect. However, considering the Kortweg-Moens approximation [13,14], the material would have to be very compliant and then structural integrity issues have to be taken into account. This fact limits the available options regarding tube materials when for example high pressure hydraulics are considered or extensive hosing effects might manifest.

**Figure 1.**
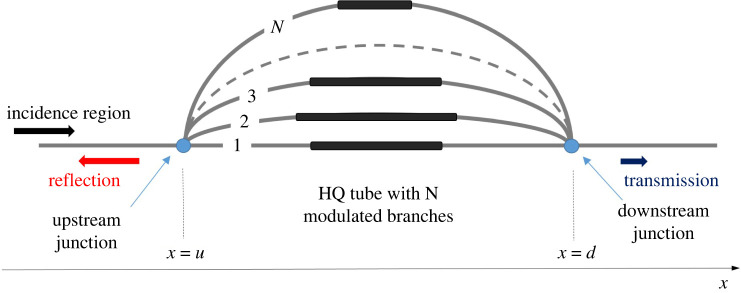
Herschel-Quincke tube with modulated branches. Branches consist of three regions. The upstream unmodulated region, the modulated region and the downstream unmodulated region. (Online version in colour.)

A different approach could be to enhance reflection characteristics of the HQ tube by introducing modulated branches. A very recent study [15] introduces the concept of the Virtual HQ tube. In the Virtual HQ tube, a periodic array of identical resonators is embedded in one of the branches. This approach has been found in [15] to exploit periodicity and generate broadband attenuation, hence leading to very efficient silencers. The basic idea in [15], i.e. to use the properties of periodicity, is adopted in the present study as well. Only in this case is the pipe stiffness or thickness properties vary periodically. Of course, modulations of pipe stiffness or thickness can produce enhanced transmission loss even when a single pipe is used [16,17]. A key hypothesis of the present study is that synergies between pipe modulation inducing reflection and destructive interference from parallel branches could yield very good attenuation features. Hence, the concept of the Modulated Herschel-Quincke (MHQ) tube is introduced. The new concept extends results from Selamet & Easwaran [7] to include modulated branches in an *N*-duct HQ tube. Similarly to [15], the MHQ uses in certain cases periodicity (but through appropriate modulation in the pipe properties) allowing for branches where the celerity is a function of the spatial variable. It therefore introduces complex reflection phenomena and additional Transmission Loss bands. The MHQ tube concept is depicted in figure 1. Some, or all, of the branches include modulated pipe segments. In the modulated segments pulse propagation velocity differs from that of the standard ducts and can be a function of the spatial variable. Incident waves reach the Upstream Junction and enter the branches. Pulses in each branch propagate with different wavenumbers and meet again at the Downstream Junction. By appropriately tuning the number of branches or their modulation, destructive interference occurs. Consequently, there is no pulse propagation after the Downstream Junction. Energy conservation then dictates that the pulse is fully reflected and the tube acts as a filter for the transmission region.

The paper is organized as follows. Section 2 presents the governing equations for long wavelength pressure and volumetric flow rate pulses in pipes with variable celerity characteristics. These equations constitute the basis in developing the MHQ tube theory. In §3, the transfer matrix for a modulated branch is derived and three cases of modulation, namely continuously varying profiles, stratified and periodically stratified profiles are introduced. Sections 4 and 5 present the overall transfer matrix formulation and resonance condition equations for the MHQ tube, respectively. Finally, several examples that verify the derived formulaes and demonstrate the effectiveness of the MHQ tube are included in §6.

## 2. Governing equations

To study pulse reflection and transmission in modulated pipes, a pulse of unit amplitude and angular frequency *ω* is set to propagate rightwards inside a pipe filled with fluid of density *ρ*. Assuming small amplitude disturbances and low Mach numbers, the acoustic volume velocity *q* = *Au* and pressure *p* satisfy the linearized mass and momentum balance equations [18,19]

2.1 $$\frac{\partial p}{\partial t}+c^{2}\frac{\rho }{A}\frac{\partial q}{\partial x}=0$$

and

2.2 $$\frac{\partial q}{\partial t}+\frac{A}{\rho }\frac{\partial p}{\partial x}=0,$$

where *A* is the undistorted cross-section area of the pipe, *u* the velocity and

2.3 $$c^{2}=\frac{K_{\text{f}}Eh}{\rho (Eh+\chi K_{\text{f}}D)}$$

defines the Kortweg-Moens celerity *c*. Coefficient *χ* depends on the pipe Poisson's ratio and has the value *χ* = 1 − *ν*/2 when the pipe is constrained at its upstream end only, *χ* = 1 − *ν*^2^ for a pipe constrained throughout from axial movement and *χ* = 1 for a pipe constrained with expansion joints throughout (Korteweg model) [18]. In the celerity definition, *E* is the pipe material Young's modulus, *h* the pipe wall thickness, *K*_f_ the hydraulic fluid bulk modulus and *D* the internal pipe diameter. The fundamental assumption in the employed model is that wavelengths are large compared to the characteristic dimensions of the pipe cross-section, e.g. inner radius, and that the velocity profile is approximately uniform in each cross-section [19]. A modulated profile of length 2*L* is now considered. The modulation refers to the product *Eh* and is of the form *δf*(*x*), where *δ* denotes modulation amplitude and |*f*(*x*)| ≤ 1. Then it is

2.4 $$c^{2}(x)=\frac{K_{f}Eh[1+\delta f(x)]}{\rho [Eh(1+\delta f(x))+\chi K_{f}D]}=c_{\mathrm{ref}}^{2}\frac{1+\delta f(x)}{1+r\delta f(x)},$$

where *r* = *Eh*(*Eh* + *χK_f_D*)^−1^ < 1 and $c_{\mathrm{ref}}^{2}=rK_{f}/\rho$.

Eliminating *q* from (2.1) and (2.2), and assuming solutions of the form *q* = *Q*(*x*)e^−i*ω*^*^t^*, *p* = *P*(*x*)e^−i*ω*^*^t^*, a Helmholtz type equation with variable wavenumber *k*(*x*) = *ω*/*c*(*x*) occurs

2.5 $$\frac{{\text{d}}^{2}P}{\text{d}x^{2}}+k^{2}(x)P=0.$$

The general solution of (2.5) is of the form

2.6 $$P(x)=C_{1}F(x)+C_{2}G(x),$$

and from (2.2) the amplitude *Q* can be calculated as

2.7 $$iQ=\frac{A}{\omega \rho }\frac{\text{d}P}{\text{d}x}=\frac{A}{\omega \rho }\left[C_{1}\frac{\text{d}F}{\text{d}x}+C_{2}\frac{\text{d}G}{\text{d}x}\right].$$

Finally, to facilitate analytical solutions for certain cases of complex celerity profiles, mild modulations will be introduced. In such cases it is *δ* ≪ 1 and an approximation of the wavenumber in (2.5), using McLaurin's expansion [1 + *δf*(*x*)]^−1^ = 1 − *δf*(*x*) + *O*(*δ*^2^), is

2.8 $$k^{2}(x)=\frac{\omega^{2}}{c^{2}(x)}=\frac{\omega^{2}}{c_{\text{ref}}^{2}}\frac{1+r\delta f(x)}{1+\delta f(x)}\approx \frac{\omega^{2}}{c_{\text{ref}}^{2}}[1-\varepsilon f(x)],$$

where ε = (1 − *r*)*δ*. The case of unmodulated profiles corresponds to ε = 0. Analytical solutions in terms of *F*, *G* for several profiles *f*(*x*) of mild modulation can be found in appendix A.

## 3. Transfer matrix for a modulated branch

In this section, the transfer matrix relating pressure and acoustic volume velocity upstream and downstream of the *n*th modulated branch will be derived. Transfer-matrix formulations, along with other approaches such as the mobility-matrix, facilitate greatly pulse propagation analysis in one-dimensional circuits [7,20] and have been used excessively to study the HQ tube [7,8].

**Figure 2.**
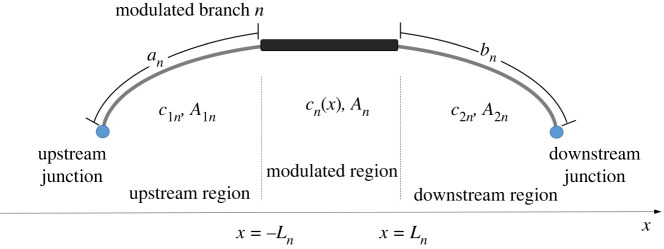
Length, celerity and cross-section area variation along a modulated branch. Properties are constant at the upstream and downstream region, while celerity is a function of the spatial variable in the modulated region. (Online version in colour.)

Each branch of the MHQ tube is composed of three regions, as shown in figure 2. First there is the upstream region with branch length *a_n_*, internal pipe cross-section area *A*_1*n*_ and constant celerity *c*_1*n*_. Adjacent to that is the modulated region defined in the interval *x* = −*L_n_* to *x* = −*L_n_* and with internal pipe cross-section *A_n_*. Celerity *c_n_*(*x*) within this region is a function of the spatial variable. After the modulated region follows the downstream part of the branch with total length *b_n_*, cross-section area *A*_2*n*_ and celerity *c*_2*n*_. In the upstream and downstream region it is *ωa_n_*/*c*_1*n*_ = *k*_1*n*_*a_n_* and *ωb_n_*/*c*_2*n*_ = *k*_2*n*_*b_n_* respectively. Furthermore, the ratios $\Xi_{1n}=\rho c_{1n}/A_{1n}$ and $\Xi_{2n}=\rho c_{2n}/A_{2n}$ for the upstream and downstream region, respectively, are introduced.

The transfer matrix equation relating *P* and *Q* in branch *n* from the upstream to the downstream junction is

3.1 $$\left[\begin{array}{c} P_{d}^{\left(n\right)}\\ iQ_{d}^{\left(n\right)} \end{array}\right]=\left[\begin{array}{cc} T_{11}^{\left(n\right)} & T_{12}^{\left(n\right)}\\ T_{21}^{\left(n\right)} & T_{22}^{\left(n\right)} \end{array}\right]\left[\begin{array}{c} P_{u}^{\left(n\right)}\\ iQ_{u}^{\left(n\right)} \end{array}\right]={\text{T}}_{n}\left[\begin{array}{c} P_{u}^{\left(n\right)}\\ iQ_{u}^{\left(n\right)} \end{array}\right]={\text{D}}_{n}{\text{M}}_{n}{\text{U}}_{n}\left[\begin{array}{c} P_{u}^{\left(n\right)}\\ iQ_{u}^{\left(n\right)} \end{array}\right],$$

where **T***_n_*, the overall transfer matrix for branch *n*, is calculated using the transfer matrices for the upstream region **U***_n_* and downstream region **D***_n_*

3.2 $$\begin{array}{rl} {\text{U}}_{n} & \;=\left[\begin{array}{cc} \cos(k_{1n}a_{n}) & \Xi_{1n}\sin(k_{1n}a_{n})\\ -\Xi_{1n}^{-1}\sin(k_{1n}a_{n}) & \cos(k_{1n}a_{n}) \end{array}\right]\\ {\text{D}}_{n} & \;=\left[\begin{array}{cc} \cos(k_{2n}b_{n}) & \Xi_{2n}\sin(k_{2n}b_{n})\\ -\Xi_{2n}^{-1}\sin(k_{2n}b_{n}) & \cos(k_{2n}b_{n}) \end{array}\right], \end{array}$$

and the transfer matrix of the modulated region

3.3 $${\text{M}}_{n}=\left[\begin{array}{cc} M_{11}^{\left(n\right)} & M_{12}^{\left(n\right)}\\ M_{21}^{\left(n\right)} & M_{22}^{\left(n\right)} \end{array}\right].$$

In the following, explicit forms of the $M_{ij}^{\left(n\right)}$coefficients in (3.3) for the case of continuously modulated profiles, stratified profiles and periodically stratified profiles will be presented. The transfer matrices defined in (3.1), (3.2) and (3.3) are unimodular, that is their determinants satisfy $\det{\text{D}}_{n}=\det{\text{M}}_{n}=\det{\text{U}}_{n}=1$ [21].

### (a) Continuously modulated profile

With reference to equation (2.5) and solution (2.6) it is

3.4 $${\text{M}}_{n}=\frac{1}{W_{n}}\left[\begin{array}{cc} W_{n}(L_{n},-L_{n}) & \frac{\omega \rho }{A_{n}}\left|\begin{array}{cc} F_{n}(-L_{n}) & G_{n}(-L_{n})\\ F_{n}(L_{n}) & G_{n}(L_{n}) \end{array}\right|\\ -\frac{A_{n}}{\omega \rho }\left|\begin{array}{cc} {F^{\prime }}_{n}(-L_{n}) & {G^{\prime }}_{n}(-L_{n})\\ {F^{\prime }}_{n}(L_{n}) & {G^{\prime }}_{n}(L_{n}) \end{array}\right| & W_{n}(-L_{n},L_{n}) \end{array}\right],$$

where

3.5 $$W_{n}(\xi ,\eta )=\left|\begin{array}{cc} F_{n}(\xi ) & G_{n}(\xi )\\ {F^{\prime }}_{n}(\eta ) & {G^{\prime }}_{n}(\eta ) \end{array}\right|=F_{n}(\xi )G_{n}^{\prime }(\eta )-F_{n}^{\prime }(\eta )G_{n}(\xi ).$$

For *ξ* = *η* it is *W_n_*(*ξ*, *ξ*) = *W_n_*(*η*, *η*) = *W_n_*. In this case, *W_n_* is the Wronskian of the two linearly independent solutions of (2.5), i.e. *F*, *G*. Because of linear independence and the governing equation form this quantity is constant and different than zero [22].

### (b) Stratified and periodically stratified profile

Assuming a modulated profile consisting of *m* layers, each one with transfer matrix **M***_j_*, the overall transfer matrix for the modulated region is ${\text{M}}_{n}=\prod_{j=1}^{m}{\text{M}}_{j}$ [22]. This product formula can be used in conjunction with piecewise constant stratification to approximate more complex celerity profiles [23–25]. A very important special case of (3.9) is when the modulation profile consists of a periodic cell repeated *m* times within the modulated region. The transfer matrix of the periodic cell in branch *n* is assumed to have the general form

3.6 $${\text{M}}_{p}=\left[\begin{array}{cc} \mu_{11} & \mu_{12}\\ \mu_{21} & \mu_{22} \end{array}\right],$$

and to be unimodular. Then, it is [20,24,25]

3.7 $${\text{M}}_{n}={\left({\text{M}}_{p}\right)}^{m}=\left[\begin{array}{cc} \mu_{11}U_{m-1}(\mu )-U_{m-2}(\mu ) & \mu_{12}U_{m-1}(\mu )\\ \mu_{21}U_{m-1}(\mu ) & \mu_{22}U_{m-1}(\mu )-U_{m-2}(\mu ) \end{array}\right],$$

where *μ* = (*μ*_11_ + *μ*_22_)/2 and *U_m_*(*μ*) is the *m*th degree Chebyshev polynomial of the second kind. Calculations are facilitated by the recursion relation

3.8 $$U_{m}(\mu )=2\mu U_{m-1}(\mu )-U_{m-2}(\mu )\;\mathrm{for}\;m\geq 2,$$

with *U*_0_(*μ*) = 1, *U*_1_(*μ*) = 2*μ* [26].

## 4. Junction conditions

To formulate the total transfer matrix equation for the MHQ tube, continuity of pressure and volumetric flux rate must be applied at the upstream and downstream junctions. In the incidence region, with cross-section area *A_I_* and celerity *c_I_*, a pressure pulse of unit amplitude propagating rightwards is considered. Then, at the upstream junction location *x* = *u*, the state variables are

4.1 $$P_{I}={\text{e}}^{\text{i}k_{I}u}+Re^{-\text{i}k_{I}u}\;\text{and}\;Q_{I}=\Xi_{I}^{-1}({\text{e}}^{\text{i}k_{I}u}-Re^{-\text{i}k_{I}u}),$$

where *k_I_* = *ω*/*c_I_*, $\Xi_{I}=\rho c_{I}/A_{I}$ and *R* is the reflection coefficient. Junction conditions upstream are

4.2 $$Q_{I}=\sum_{n=1}^{N}Q_{u}^{\left(n\right)}\;\text{and}\;P_{I}=P_{u}^{\left(n\right)},\;\text{for}\;n=1,2,\ldots N.$$

At the transmission region, with cross-section area *A_T_* and celerity *c_T_*, the transmitted pulse propagates rightwards and no reflection occurs. The state variables at the downstream junction *x* = *d* are

4.3 $$P_{T}=T{\text{e}}^{\text{i}k_{T}d}\;\text{and}\;Q_{T}=\Xi_{T}^{-1}T{\text{e}}^{\text{i}k_{T}d},$$

where *k_T_* = *ω*/*c_T_*, $\Xi_{T}=\rho c_{T}/A_{T}$ and *T* is the transmission coefficient. Junction conditions downstream are

4.4 $$Q_{T}=\sum_{n=1}^{N}Q_{d}^{\left(n\right)}\;\text{and}\;P_{T}=P_{d}^{\left(n\right)}\;\text{for}\;n=1,2,\ldots ,N.$$

Applying the junction conditions results in an overall transfer matrix for the Herschel-Quincke tube with modulated branches that relate state variables at the upstream and downstream junction

4.5 $$\left[\begin{array}{c} P_{T}\\ iQ_{T} \end{array}\right]=\frac{1}{\sum_{n=1}^{N}\left(1/T_{12}^{\left(n\right)}\right)}\left[\begin{array}{cc} T_{11} & T_{12}\\ T_{21} & T_{22} \end{array}\right]\left[\begin{array}{c} P_{I}\\ iQ_{I} \end{array}\right]=T\left[\begin{array}{c} P_{I}\\ iQ_{I} \end{array}\right]$$

where, using also the fact that $\det{\text{T}}_{n}=1$, the diagonal and off-diagonal elements are

4.6a $$T_{11}=\sum_{n=1}^{N}\left(T_{11}^{\left(n\right)}/T_{12}^{\left(n\right)}\right),T_{22}=\sum_{n=1}^{N}\left(T_{22}^{\left(n\right)}/T_{12}^{\left(n\right)}\right)$$

and

4.6b $$T_{12}=-i,\;T_{21}=-i\left\{{\left[\sum_{n=1}^{N}\left(1/T_{12}^{\left(n\right)}\right)\right]}^{2}-\sum_{n=1}^{N}\left(T_{11}^{\left(n\right)}/T_{12}^{\left(n\right)}\right)\cdot \sum_{n=1}^{N}\left(T_{22}^{\left(n\right)}/T_{12}^{\left(n\right)}\right)\right\}.$$

It can be verified that detT=1, and after straightforward calculations

4.7 $$R=\frac{-\Xi_{T}^{-1}T_{11}-{\left(\Xi_{T}\Xi_{I}\right)}^{-1}T_{12}+T_{21}+\Xi_{I}^{-1}T_{22}}{\Xi_{T}^{-1}T_{11}-{\left(\Xi_{T}\Xi_{I}\right)}^{-1}T_{12}-T_{21}+\Xi_{I}^{-1}T_{22}}e^{2ik_{I}u}$$

and

4.8 $$T=\frac{2\Xi_{I}^{-1}}{\Xi_{T}^{-1}T_{11}-{\left(\Xi_{T}\Xi_{I}\right)}^{-1}T_{12}-T_{21}+\Xi_{I}^{-1}T_{22}}{\text{e}}^{\text{i}(k_{I}u-k_{T}d)}.$$

Transmission Loss (TL) is defined as

4.9 $$\text{TL}=20\log_{10}\left|\frac{1}{T}\right|=20\log_{10}\left|\frac{\Xi_{T}^{-1}(\Xi_{I}T_{11}-T_{12})}{2}-\frac{\left(\Xi_{I}T_{21}-T_{22}\right)}{2}\right|.$$

It is noted that in the present model losses and higher order mode effects at junctions are not considered.

## 5. Resonance conditions

Equations (4.6*a*) and (4.6*b*) imply that resonance conditions occur when

5.1 $$\Omega_{N}=\sum_{n=1}^{N}\left(\frac{1}{T_{12}^{\left(n\right)}}\right)=0.$$

Element $T_{12}^{\left(n\right)}$ of the overall transfer matrix for branch *n* can be calculated from ${\text{T}}_{n}={\text{D}}_{n}{\text{M}}_{n}{\text{U}}_{n}$. For most practical applications the upstream and downstream region properties and length will be the same, i.e. $\Xi_{1n}=\Xi_{2n}=\Xi_{n}$ and *k*_1*n*_*a_n_* = *k*_2*n*_*b_n_* = *k_n_*ℓ*_n_*. If at the same time in the modulated region it is $M_{11}^{\left(n\right)}=M_{22}^{\left(n\right)}=M_{\;}^{\left(n\right)}$, formula (5.1) becomes

5.2 $$\Omega_{N}=\sum_{n=1}^{n}\frac{\Xi_{n}^{-1}}{M_{\;}^{\left(n\right)}\sin(2k_{n}\ell_{n})+\Phi^{\left(n\right)}\cos(2k_{n}\ell_{n})+\Psi^{\left(n\right)}}=0,$$

where $\Phi^{\left(n\right)}=\left(\Xi_{n}^{-1}M_{12}^{\left(n\right)}-\Xi_{n}M_{21}^{\left(n\right)}\right)/2$ and $\Psi^{\left(n\right)}=\left(\Xi_{n}^{-1}M_{12}^{\left(n\right)}+\Xi_{n}M_{21}^{\left(n\right)}\right)/2$.

In the restricted case of unmodulated branches, where even the modulated region has the same properties, it is Ψ^(*n*)^ = 0, Φ^(*n*)^ = sin (*k_n_L_n_*) and $\Xi_{n}=\rho c_{n}/A_{n}$. Then, equation (5.2), using trigonometric identities for the sine and cosine of sums reduces to the condition for resonance derived in [7].

5.3 $$\Omega_{N}=\sum_{n=1}^{N}\frac{A_{n}}{c_{n}\sin[2k_{n}(\ell_{n}+L_{n})]}=0.$$

Equations (5.1) and (5.2) generalize (5.3) in the case of modulated branches. Therefore, they feature more degrees of freedom available for calibration to achieve full reflection conditions at specific frequency bands. This attribute could be very useful when design constraints, such as branch length, number of branches and material properties (e.g. strength) are taken into account. Consequently, the option of modulated braches could facilitate spurious pulse filtering based on the HQ tube concept in very challenging designs and advanced applications. Furthermore, it could increase the number of frequency bands where high Transmission Loss occurs with respect to both band location and band width. This aspect could be useful in hydraulic circuits with positive displacement pumps for pressure ripple attenuation. In such applications, HQ tubes are not typically considered due to the narrow banded attenuation they produce [27].

## 6. Results and discussion

In this section, the theory and formulaes for the MHQ tube will be applied to the analysis and design of configurations that maximize Transmission Loss at specific frequency bands. Three examples will be presented. The first one is a verification case, where the newly derived formulaes will be applied to benchmark examples from previous studies on HQ tube performance. The second example, in §§6b, introduces an MHQ tube with a single periodically modulated parallel branch. Finally, example 6.3 considers the same periodically modulated profile but with two parallel branches, increasing the total number of branches of the MHQ tube to three.

### (a) Verification example

This first example aims to serve as verification of the formulae derived for the MHQ tube and at the same time highlight some significant features that modulation inflicts on Transmission Loss characteristics of the HQ tube. To verify the formulae a benchmark case from the relevant literature will be used. The approach adopted is to consider a configuration already analysed by other researchers and allow for the parallel branch to be mildly modulated. When the modulation amplitude ε tends to zero, the results of the unmodulated HQ tube are to be reproduced. For mild modulations, solution properties, such as energy conservation, will be verified and a comparative study of the TL diagrams for small values of the amplitude parameter will be conducted.

**Figure 3.**
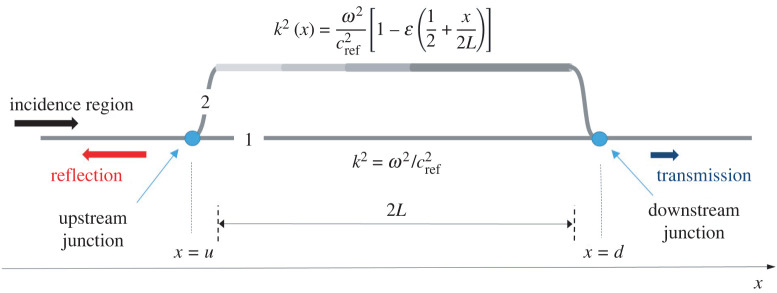
MHQ tube with modulated parallel branch. The modulated branch includes a segment of length 2*L* continuously varying celerity. Properties of the configuration are given in table 1. (Online version in colour.)

The MHQ tube in figure 3 is considered. The main branch, termed Branch 1, is unmodulated while the parallel branch, termed Branch 2, is mildly modulated according to the formula (2.8). Parameters of the MHQ tube are given in table 1. All dimensions and the reference celerity *c_ref_* = 343 m s^−1^ are selected to correspond to Configuration 1 from the 1994 study of Selamet & Dickey [6] (page 3183). This configuration has also been validated using experimental and computational results in [6]. In the present example, for the modulated branch the mildly modulated profile *f*(*x*) = 1/2 + *x*/2*L* is selected. The linearly independent solutions of equation (2.5), needed to formulate transfer matrix (3.6), are actually the Airy functions and the relevant arguments can be found in Appendix A. The incidence and transmission region celerity is *c_I_* = *c_T_* = *c*_ref_ = 343 m s^−1^. The internal tube diameter in both these regions is *d_I_* = *d_T_* = 4.859 cm. Formula (2.8) for the mildly modulated wavenumber indicates that the results for modulation amplitude ε → 0 should approach the TL diagram in figure 10 of Selamet & Dickey.

**Table 1.** Parameters for the MHQ tube in example 6.1.

	upstream region			modulated region			downstream region		
	$a_{n}$	$c_{1n}$	$d_{1n}$	$2L_{n}$	$c_{n}$	$d_{n}$	$b_{n}$	$c_{2n}$	$d_{2n}$
	(cm)	$\left(m\;s^{-1}\right)$	(cm)	(cm)	$\left(m\;s^{-1}\right)$	(cm)	(cm)	$\left(m\;s^{-1}\right)$	(cm)
Branch 1									
*n* = 1	4	343	4.859	31.85	343	4.859	4	343	4.859
Branch 2									
*n* = 2 (modulated)	23.3	343	4.674	31.85	$\frac{343}{\sqrt{1-\varepsilon f(x)}}$	4.674	23.3	343	4.674

**Figure 4.**
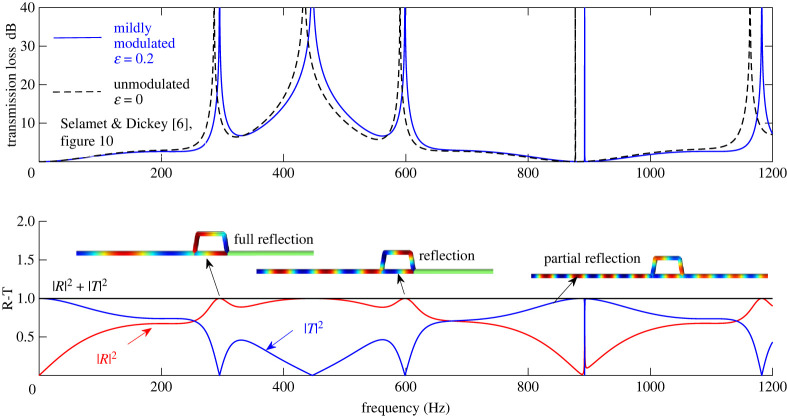
Transmission Loss (upper plot) of the MHQ tube in figure 3. Properties of the configuration are given in table 1. For very mild modulations the TL characteristics (blue solid line) tend to coincide with the results of Selamet & Dickey (black dotted line). Reflection-Transmission diagram (lower plot) for the same configuration and comparison with COMSOL solutions for three frequencies. (Online version in colour.)

In figure 4, the Transmission Loss (TL) and Reflection-Transmission coefficients for modulation amplitude ε = 0.2 are plotted. The very mild modulation is found to shift the TL spikes towards higher frequencies. Indeed, as ε → 0, the effect of modulation is reduced and the results of the MHQ tube tend to coincide with the results of Selamet & Dickey (black dashed line). The Reflection-Transmission (R-T) diagram corresponding to the same example demonstrates that |*R*|^2^ + |*T*|^2^ for the MHQ tube is equal to one for all frequencies, since due to energy conservation this sum should equal the amplitude of the incoming pulse. Furthermore, for two characteristic frequencies of full reflection (approx. 295 and 599 Hz) and a frequency of partial reflection (840 Hz), the MHQ tube circuit has been simulated using COMSOL Pipe Flow Module. Graphical results for these three frequencies are embedded in the R-T diagram of figure 4. Results are in perfect agreement with the predictions of the analytical solution, COMSOL yielding zero pressure pulse in the transmission branch when the response in frequencies of full reflection is simulated.

### (b) Parallel branch with periodic modulation

**Figure 5.**
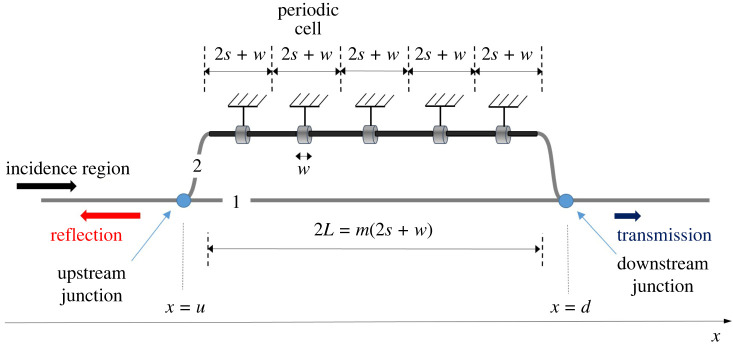
MHQ tube with one modulated parallel branch. The modulated branch includes a segment of length 2*L* with lower celerity and periodically placed stiffeners of width *w* that increase celerity locally. Properties of the configuration are given in table 2. (Online version in colour.)

The MHQ configuration in figure 5 is considered. The tube has the main, unmodulated, branch denoted as Branch 1 and one periodically modulated parallel branch (Branch 2). The modulated branch between *x* = −*L* and *x* = *L* has a pipe with reduced stiffness, and hence reduced celerity denoted as *c*_s_. To avoid hosing effects, stiffeners of width *w* are placed along the modulated region. At the stiffener locations, the celerity is increased to *c_w_*. The distance between two successive stiffeners is 2*s*. The first and last stiffener are placed at distance *s* from the left- and right-hand side ends of the flexible pipe region, respectively. A periodic cell of length ℓ = 2*s* + *w* is thus defined. Figure 5 depicts the case of five such periodic cells. The approach presented in sub-section 3b for periodic stratifications will be adopted in the following. Denoting ${\text{M}}_{s}$ and ${\text{M}}_{w}$ the transfer matrix for part *s* and part *w* respectively, the transfer matrix of the periodic cell is

6.1 $${\text{M}}_{\text{p}}={\text{M}}_{\text{s}}{\text{M}}_{\text{w}}{\text{M}}_{\text{s}}=\left[\begin{array}{cc} \mu_{11} & \mu_{12}\\ \mu_{21} & \mu_{22} \end{array}\right],$$

with

6.2a $$\mu_{11}=\mu_{22}=\mu =\cos\left(\frac{2\omega s}{c_{s}}+\frac{\omega w}{c_{w}}\right)-\Lambda \sin\left(\frac{\omega w}{c_{w}}\right)\sin\left(\frac{2\omega s}{c_{s}}\right),$$

6.2b $$\mu_{12}=\Xi_{s}\left\{\sin\left(\frac{2\omega s}{c_{s}}+\frac{\omega w}{c_{w}}\right)-\sin\left(\frac{\omega w}{c_{w}}\right)\left[K-\Lambda \cos\left(\frac{2\omega s}{c_{s}}\right)\right]\right\}$$

6.2c $$\text{and}\;\mu_{21}=-\Xi_{s}^{-1}\left\{\sin\left(\frac{2\omega s}{c_{s}}\right)+\frac{\omega w}{c_{w}}+\sin\left(\frac{\omega w}{c_{w}}\right)\left[K+\Lambda \cos\left(\frac{2\omega s}{c_{s}}\right)\right]\right\},$$

where $\Xi_{s}=\rho c_{s}/A_{n}$, $\Xi_{w}=\rho c_{w}/A_{n}$ and $K=\Xi_{s}^{2}-\Xi_{w}^{2}/2\Xi_{s}\Xi_{w}$, $\Lambda ={\left(\Xi_{s}-\Xi_{w}\right)}^{2}/2\Xi_{s}\Xi_{w}$.

The modulated region transfer matrix for *m* periodic cells can now be calculated from equation (3.8). The upstream and downstream region have same properties and length. Since *μ*_11_ = *μ*_22_, (3.8) implies that $M_{11}^{\left(n\right)}=M_{22}^{\left(n\right)}=M^{\left(n\right)}$. Therefore resonance frequencies correspond to roots of (5.2).

A specific configuration is considered, where the incidence and transmission region celerity is set to *c_I_* = *c_T_* = 1000 m s^−1^. The internal tube diameter in both these regions is *d_I_* = *d_T_* = 0.02 m. The upstream junction is located at *x* = *u* = −0.05 m and the downstream junction is located at *x* = *d* = 1.05 m. With reference to figure 5, the length of modulation is selected to be 2*L* = 1 m. The unmodulated branch, indicated in the following as Branch 1, has therefore total length of 1.1 m. Celerity *c*_1*n*_, *c*_2*n*_, *c_n_*, length *a_n_*, *L_n_*, *b_n_* and pipe diameter *d*_1*n*_, *d_n_*, *d*_2*n*_ values for *n* = 1 (Branch 1) and *n* = 2 (Branch 2) in the upstream, modulated and downstream region are summarized in table 2. The stiffener width in the modulated branch is *w* = 0.05 m. The transmission loss (TL) characteristics of the modulated HQ tube will be compared to those of a HQ with the same geometric characteristics (lengths, number of branches, pipe diameter) but with constant celerity along all the HQ components equal to that of the incidence and transmission region, i.e. *c* = 1000 m s^−1^. This reference HQ tube will be referred to as unmodulated.

**Table 2.** Parameters for the MHQ tube in example 6.2.

	upstream region			modulated region			downstream region		
	$a_{n}$	$c_{1n}$	$d_{1n}$	$2L_{n}$	$c_{n}$	$d_{n}$	$b_{n}$	$c_{2n}$	$d_{2n}$
	(m)	$\left(m\;s^{-1}\right)$	(m)	(m)	$\left(m\;s^{-1}\right)$	(m)	(m)	$\left(m\;s^{-1}\right)$	(m)
Branch 1									
*n* = 1	0.05	1000	0.01	1	1000	0.01	0.05	1000	0.01
Branch 2					*c_s_* = 500				
*n* = 2 (modulated)	0.2	1000	0.01	1	*c_w_* = 1000	0.01	0.2	1000	0.01

**Figure 6.**
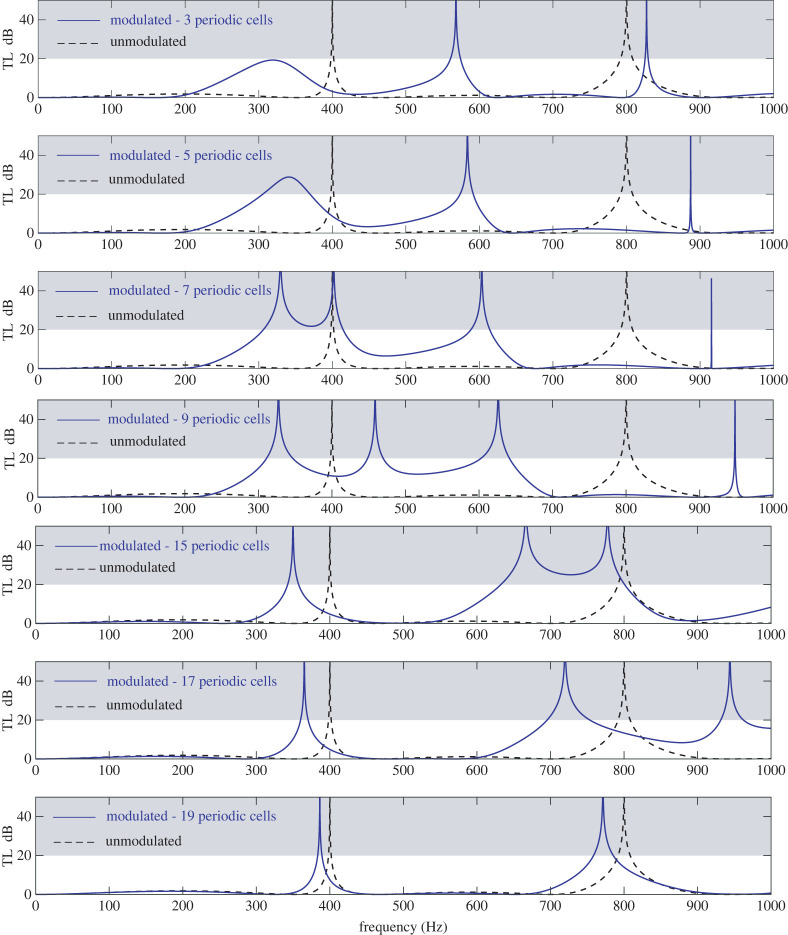
Transmission Loss of an unmodulated HQ tube (dashed black line) versus the modulated MHQ tube depicted in figure 5 (solid blue line) with increasing number of periodic cells. Properties of the configuration are given in table 2. (Online version in colour.)

Transmission Loss characteristics for the modulated and unmodulated version of the HQ tube in figure 5 are summarized in figure 6. Analysis in the frequency range up to 1000 Hz is presented for increasing number of periodic cells in the modulated HQ tube. Efficient transmission loss is set to be achieved for values higher than 20 dB. This regime is indicated using the shaded area in figure 6. The solid blue line represents performance of the modulated HQ tube while the dashed black line represents the performance of the unmodulated tube. The case of three periodic cells is found to introduce a reflection tongue (appearing as a hump in the first subplot) in the frequency range from approximately 200 to 400 Hz. At the same time, transmission loss spikes are shifted to higher frequencies compared to the unmodulated tube. Increasing the number of periodic cells to five leads to a further, though very slight, shifting toward higher frequencies. At the same time the reflection tongue amplitude increases significantly featuring now a wide band (approx. from 320 to 370 Hz) above the 20 dB threshold. For seven periodic cells, the hump splits in two TL spikes with a band of about 100 Hz being over 20 dB. Further increase of the periodic cells shifts the TL spikes to higher frequencies. For nineteen periodic cells the TL diagram of the modulated HQ resembles closely the one of the unmodulated one. Since *c_w_* = 1000 m s^−1^ and the stiffener width is *w* = 0.05 m, the use of twenty periodic cells will lead to the whole modulated region having celerity equal to the unmodulated tube.

**Figure 7.**
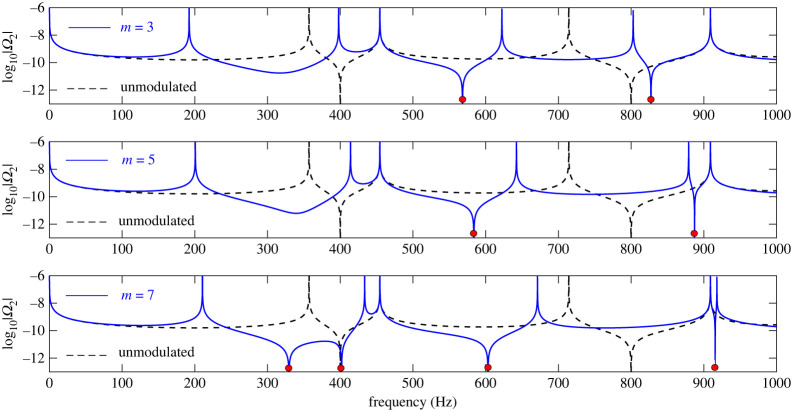
Roots of Ω*_N_* for the MHQ tube (blue solid line) according to (5.2), plotted as spikes of log _10_|Ω*_N_*| and indicated using red dots. Unmodulated HQ tube result indicated using black dashed line. (Online version in colour.)

The predictions of formula (5.2) regarding resonant frequencies of the tube are plotted in figure 7 for the case of 3, 5 and 7 periodic cells. For demonstration purposes, the logarithm of |Ω*_N_*| is plotted. Then, roots of Ω*_N_* correspond to spikes tending to minus infinity (indicated by red dots in figure 7). Finally, it is worth mentioning that effects of periodicity can also be implemented using continuously modulated profiles, such as the ones resulting from functionally graded materials. The formulaes derived in this study can facilitate simple analytical solutions even in such cases. In Appendix A, the second and third row of table 3 present functions *F*, *G* for a simple sinusoidal profile (corresponding to periodic stiffening and softening of the modulated tube) and a profile corresponding to the square of a sinusoidal variation representing periodic stiffening or softening depending on the sign of the profile.

### (c) Double parallel branch with periodic modulation

**Figure 8.**
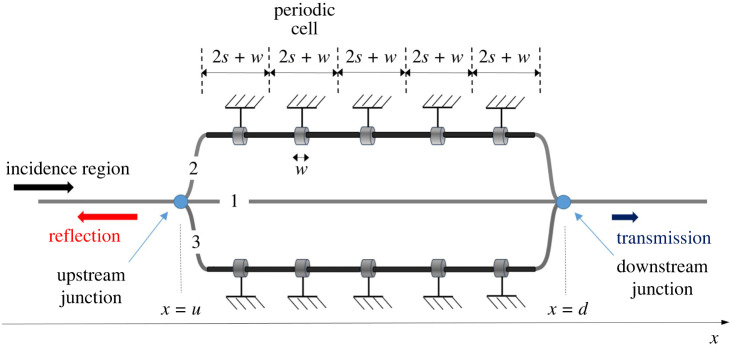
MHQ tube with two modulated parallel branches. The modulated branches include a segment of length 2*L* with lower celerity and periodically placed stiffeners of width *w* that increase celerity locally. Each branch is identical to the one in example 6.2. (Online version in colour.)

The periodic cell modulation introduced in example 6.2 is considered again but is now applied to two parallel branches. The MHQ tube in this case has, therefore, a total of three branches and is shown in figure 8. All parameters are identical to the ones used in §6b with only the number of modulated branches changing to two. A parametric study on the Transmission Loss (TL) is conducted with respect to increasing number of periodic cells.

**Figure 9.**
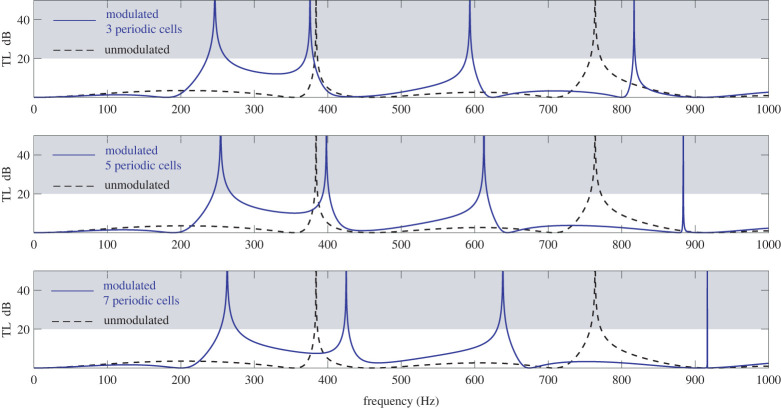
MHQ tube with two parallel modulated branches, as depicted in figure 8, for different numbers of periodic cells. Transmission Loss (TL) spikes increase with modulation and first spike appears in lower frequencies. Properties of the modulated branches are the same as in example 6.2. (Online version in colour.)

Figure 9 demonstrates the cases of 3, 5 and 7 periodic cells. Compared to the unmodulated HQ tube, the case of two parallel modulated branches produces two more TL spikes even when only three or five periodic cells are used. Furthermore, the first transmission loss spikes appear now in lower frequencies compared to the case of the modulated HQ tube with only one parallel branch.

**Figure 10.**
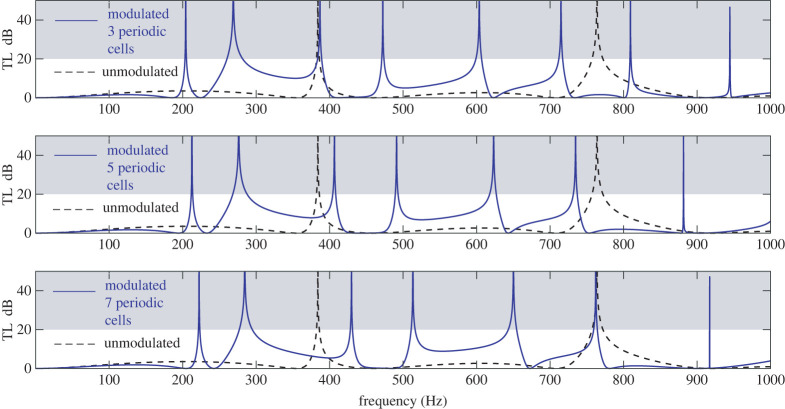
MHQ tube with two parallel modulated branches as depicted in figure 8. Celerity now differs in the two parallel branches. It is *c*_s_ = 500 m s^−1^ in one modulated branch and *c*_s_ = 600 m s^−1^ in the second. Stiffener celerity is *c*_w_ = 1000 m s^−1^ in both branches. (Online version in colour.)

A slight modification to the celerity of the modulated region in only one of the two parallel branches is investigated too. In figure 10, the modulated HQ tube with two parallel branches is considered again, only the celerity *c*_s_ in one of the two modulated branches (Branch 3) is changed from 500 m s^−1^ to 600 m s^−1^. This slight modification produces a significant change in the TL diagram. The number of TL spikes for three periodic cells now doubles from four to eight, while the first spike appears in even lower frequency. This configuration type, with appropriate calibration, could be useful in cases where several different frequency components need to be attenuated simultaneously, such as the pressure ripples from piston pumps in hydraulic circuits [27].

## 7. Conclusion

The concept of the modulated Herschel-Quincke tube (MHQ) is introduced. The MHQ tube allows for parallel branches with variation of the celerity profile along them. Closed form expressions for the prediction of Transmission Loss and Resonant frequencies in the presence of continuously varying or stratified profiles are derived. The results are verified and the effectiveness of the MHQ in generating multiple or broadband transmission loss bands is demonstrated using configurations with periodically stratified profiles. The modulated profiles of the parallel branches introduce more design parameters in the HQ tube concept and hence more flexibility in achieving enhanced reflection without resorting to branches of extensive length. This attribute could be potentially useful in applications where space limitations are imposed. The present analysis targets high-pressure hydraulics in particular. In such applications, cross-section deformation and hence influence of pipe stiffness on the speed of sound becomes important. Therefore, the MHQ tube concept could potentially be applied for pressure ripple noise reduction [27], achieving more broadband attenuation compared to the standard HQ tube. Future aims include extending the MHQ tube theory to periodically modulated tubes that feature more deformation modes [28] and taking into account mean-flow as well as pipe axial tension effects [29].

## Data Availability

This article has no additional data.

## Appendix A

In the following table, table 3, specific cases of modulated profiles *f*(*x*) for the second order differential equation (2.5), i.e.

A 1 $$\frac{{\text{d}}^{2}P}{\text{d}x^{2}}+\frac{\omega^{2}}{c_{\mathrm{ref}}^{2}}[1-\varepsilon f(x)]P=0,\;-L<x<L,$$

with |*f*(*x*)| ≤ 1 and ε ≪ 1.

**Table 3.** Different profiles and governing equations for the modulated region ( − *L*, *L*).

*f*(*x*)	equation type and standard form	solution *P*(*x*) = *C*_1_*F*(*x*) + *C*_2_*G*(*x*)
$\pm \frac{x}{2L}+\frac{1}{2}$	Airy	*F*(*x*) = Ai(*α*^1/3^*x* + *α*^−2/3^*β*)
	$\frac{d^{2}P}{d\xi^{2}}-(\alpha \xi +\beta )P=0$	*G*(*x*) = Bi(*α*^1/3^*x* + *α*^−2/3^*β*)
		α=±εω22Lcrefaaa2 , $\beta =-\frac{\omega^{2}}{c_{\mathrm{ref}}^{2}}\left(1-\frac{\varepsilon }{2}\right)$
$\sin\left(\frac{m\pi x}{L}\right)$	Mathieu	$F(x)=M_{1}\left(a,q,\frac{m\pi x}{2L}-\frac{\pi }{4}\right)$
	$\frac{d^{2}P}{d\xi^{2}}+[a-2q\cos(2\xi )]P=0$	$G(x)=M_{2}\left(a,q,\frac{m\pi x}{2L}-\frac{\pi }{4}\right)$
		a=4ω2L2m2π2crefaaa2, q=2εω2L2m2π2cref2
$\pm \sin^{2}\left(\frac{m\pi x}{L}\right)$	Mathieu	$F(x)=M_{1}\left(a,q,\frac{m\pi x}{L}\right)$
	$\frac{d^{2}P}{d\xi^{2}}+[a-2q\cos(2\xi )]P=0$	$G(x)=M_{2}\left(a,q,\frac{m\pi x}{L}\right)$
		a=ω2L2m2π2crefaaa2(1∓ε2), q=εω2L24m2π2cref2
